# An Online Intervention Comparing a Very Low-Carbohydrate Ketogenic Diet and Lifestyle Recommendations Versus a Plate Method Diet in Overweight Individuals With Type 2 Diabetes: A Randomized Controlled Trial

**DOI:** 10.2196/jmir.5806

**Published:** 2017-02-13

**Authors:** Laura R Saslow, Ashley E Mason, Sarah Kim, Veronica Goldman, Robert Ploutz-Snyder, Hovig Bayandorian, Jennifer Daubenmier, Frederick M Hecht, Judith T Moskowitz

**Affiliations:** ^1^ Department of Health Behavior and Biological Sciences School of Nursing University of Michigan Ann Arbor, MI United States; ^2^ Osher Center for Integrative Medicine School of Medicine University of California, San Francisco San Francisco, CA United States; ^3^ School of Medicine University of California, San Francisco San Francisco, CA United States; ^4^ Applied Biostatistics Laboratory School of Nursing University of Michigan Ann Arbor, MI United States; ^5^ University of California, Berkeley Berkeley, CA United States; ^6^ Institute of Holistic Health Department of Health Education San Francisco State University San Francisco, CA United States; ^7^ Feinberg School of Medicine Northwestern University Chicago, IL United States

**Keywords:** eHealth, diet, weight loss, type 2 diabetes mellitus

## Abstract

**Background:**

Type 2 diabetes is a prevalent, chronic disease for which diet is an integral aspect of treatment. In our previous trial, we found that recommendations to follow a very low-carbohydrate ketogenic diet and to change lifestyle factors (physical activity, sleep, positive affect, mindfulness) helped overweight people with type 2 diabetes or prediabetes improve glycemic control and lose weight. This was an in-person intervention, which could be a barrier for people without the time, flexibility, transportation, social support, and/or financial resources to attend.

**Objective:**

The aim was to determine whether an online intervention based on our previous recommendations (an ad libitum very low-carbohydrate ketogenic diet with lifestyle factors; “intervention”) or an online diet program based on the American Diabetes Associations’ “Create Your Plate” diet (“control”) would improve glycemic control and other health outcomes among overweight individuals with type 2 diabetes.

**Methods:**

In this pilot feasibility study, we randomized overweight adults (body mass index ≥25) with type 2 diabetes (glycated hemoglobin [HbA_1c_] 6.5%-9.0%) to a 32-week online intervention based on our previous recommendations (n=12) or an online diet program based around a plate method diet (n=13) to assess the impact of each intervention on glycemic control and other health outcomes. Primary and secondary outcomes were analyzed by mixed-effects linear regression to compare outcomes by group.

**Results:**

At 32 weeks, participants in the intervention group reduced their HbA_1c_ levels more (estimated marginal mean [EMM] –0.8%, 95% CI –1.1% to –0.6%) than participants in the control group (EMM –0.3%, 95% CI –0.6% to 0.0%; *P*=.002). More than half of the participants in the intervention group (6/11, 55%) lowered their HbA_1c_ to less than 6.5% versus 0% (0/8) in the control group (*P*=.02). Participants in the intervention group lost more weight (EMM –12.7 kg, 95% CI –16.1 to –9.2 kg) than participants in the control group (EMM –3.0 kg, 95% CI –7.3 to 1.3 kg; *P*<.001). A greater percentage of participants lost at least 5% of their body weight in the intervention (10/11, 90%) versus the control group (2/8, 29%; *P*=.01). Participants in the intervention group lowered their triglyceride levels (EMM –60.1 mg/dL, 95% CI –91.3 to –28.9 mg/dL) more than participants in the control group (EMM –6.2 mg/dL, 95% CI –46.0 to 33.6 mg/dL; *P*=.01). Dropout was 8% (1/12) and 46% (6/13) for the intervention and control groups, respectively (*P*=.07).

**Conclusions:**

Individuals with type 2 diabetes improved their glycemic control and lost more weight after being randomized to a very low-carbohydrate ketogenic diet and lifestyle online program rather than a conventional, low-fat diabetes diet online program. Thus, the online delivery of these very low-carbohydrate ketogenic diet and lifestyle recommendations may allow them to have a wider reach in the successful self-management of type 2 diabetes.

**Trial Registration:**

ClinicalTrials.gov NCT01967992; https://clinicaltrials.gov/ct2/show/NCT01967992 (Archived by WebCite at http://www.webcitation.org/6o0fI9Mkq)

## Introduction

Type 2 diabetes mellitus is a rapidly growing chronic disease that affects approximately 22 million people in the United States, for which diet is an integral aspect of treatment [1,2]. Data suggest that very low-carbohydrate diets [3-11], and adequate sleep and physical exercise [12-16] can improve glycemic control and reduce body weight in individuals with type 2 diabetes. Moreover, behavioral adherence strategies, including positive affect regulation and mindful eating strategies, may reduce overall stress, stress-based eating, and depressive symptoms, which can be barriers for following behavioral recommendations [17-19].

In prior research, we found that recommendations to follow a very low-carbohydrate diet and to make lifestyle changes (sleep and exercise recommendations and a package of behavioral adherence strategies based on positive affect regulation and mindfulness) were able to improve glycemic control and reduce body weight in overweight individuals with type 2 diabetes or prediabetes [20]. Although promising, this previous trial was delivered in-person, which is a significant barrier to engagement for people without the time, flexibility, transportation, social support, and/or financial resources to attend. To create a highly disseminable, evidence-based program, we adapted our in-person intervention for online delivery.

In this pilot feasibility and acceptability study, we assessed whether overweight individuals with type 2 diabetes, randomized to receive an online intervention based on our previous trial (recommendations to follow an ad libitum very low-carbohydrate ketogenic diet and other lifestyle changes), would have greater improvements in glycemic control and other health outcomes than participants randomized to a control group, an online diet program based on a plate method diet (the American Diabetes Associations’ “Create Your Plate” diet). To our knowledge, this is the first online randomized controlled trial to teach a very low-carbohydrate ketogenic diet to individuals with type 2 diabetes.

## Methods

### Participants and Procedure

We conducted a parallel-group, balanced randomization (1:1) trial, approved by the University of California, San Francisco, Institutional Review Board and registered with ClinicalTrials.gov (NCT01967992). The primary outcome measure was glycemic control, operationalized as change in glycated hemoglobin (HbA_1c_). A key secondary outcome was body weight. Exploratory outcomes were cholesterol, triglycerides, diabetes-related distress, subjective experiences of the diet, and physical side effects.

We recruited participants nationally with online ads (on Craigslist, Backpage, and Facebook), newspaper ads and articles, and radio ads. This allowed us to recruit participants from across the United States. Eligibility criteria included age 18 years or older with a body mass index of ≥25, an elevated HbA_1c_ level diagnostic of type 2 diabetes (6.5%-9%, measured by us at baseline), and regular access to the Internet. To reduce the risk of hypoglycemia, we excluded participants who were taking any diabetes medication other than metformin.

Recruitment materials directed interested participants to a study website to complete an online eligibility questionnaire. Study staff then called potentially eligible participants to assess initial eligibility and describe study procedures. For example, we assessed whether participants were taking any medications for their type 2 diabetes other than metformin. If participants then consented to the full study, they were asked to complete several assessments, all specified in the measures section.

We recruited participants who were ready to make the changes required of the intervention in order to mitigate a potentially high dropout rate. We measured readiness to undertake the intervention [21] with the following item: “If you are eligible for this study you will be asked to...cut out the kinds of cookies, cakes, pasta, pastries, bagels, rice, potatoes, and sugary fruits that some people often eat. Given the description of the dietary changes above, how prepared do you feel to make these changes?” Participants could answer the item from 1 (not at all) to 7 (very much so). Participants were eligible if they rated themselves to be prepared to begin above the midpoint of the scale (5-7). In addition, conscientious people, we reasoned, would be more likely to follow the behavior changes requested by the intervention because conscientiousness has been shown to be positively related to following beneficial health-related behaviors [22]. Participants were eligible if they rated themselves as conscientious on two items [23]: “I see myself as someone who is dependable, self-disciplined” (eligible answers were agree and strongly agree) and “I see myself as someone who is disorganized, careless” (eligible answers were disagree and strongly disagree). We used items from the Yale Food Addiction Scale [24] and the Eating Disorder Diagnostic Scale [25] to screen out participants who had the tendency to be addicted to food or binge eat.

For this study, it was not possible for the participants and staff to be masked to group allocation. Therefore, once all baseline measurements had been completed, study staff randomized participants to one of the two intervention groups by opening the next opaque envelope in a series containing the concealed sequence for randomization, which was created by a statistician using block randomization procedures, with blocks of size randomly allocated to size 2, 4, or 6.

Outcomes were measured at baseline as well as 16 and 32 weeks after baseline. We paid participants US $25 for each assessment at 16 and 32 weeks. Thus, participants could receive up to US $50 over the course of the study.

### Intervention

#### Intervention Group: Very Low-Carbohydrate Ketogenic Diet and Lifestyle Recommendations

We randomized half of participants to receive recommendations on how to eat an ad libitum very low-carbohydrate ketogenic diet, to reduce carbohydrate intake to between 20-50 grams of nonfiber carbohydrates a day with the goal of restricting carbohydrate intake to a level at which a low amount of ketone production is induced, called nutritional ketosis. In this state, the body uses fatty acids instead of carbohydrates as its primary energy source, which do not elevate glucose levels as strongly as carbohydrates [26,27]. To support dietary adherence, we mailed participants in this group urinary acetoacetate (a ketone that can be measured in urine) test kits (KetoStix, Abbott). We asked them to measure their urine for the presence of ketones at least once a week for the first few months of the program.

We also suggested that participants in the intervention group follow lifestyle recommendations, including behavioral adherence strategies aimed at increasing positive affect regulation [18] and mindful eating based largely on the Mindfulness-Based Eating Awareness Training program [19,28], using handouts and lesson content adapted from our in-person intervention. Specific topics included setting attainable goals; scheduling, noticing, and savoring positive events; developing self-compassion; practicing positive reappraisal, gratitude, and acts of kindness; being aware of one’s personal strengths; and being mindful of hunger, fullness, cravings, taste satisfaction, and triggers for overeating. Moreover, starting in week 6, the lessons discussed the importance of physical activity and sleep as well as encouraged participants to increase their level of physical activity and amount of sleep. We chose to include a comprehensive program of behavioral support in this intervention group in order to enhance the likelihood of finding an impact of our previously successful in-person program using an online format.

We emailed participants in this group new lessons weekly for the first 16 weeks and then every two weeks for the remaining 16 weeks of the study. The lessons in the first 16 weeks included short videos created for the study about all of the study components (about 5-15 minutes long, with audio narration over videos with white text, images, and a black background), with printable handouts and links to online resources, such as recipes and recipe books. The lessons in the last 16 weeks did not include study-specific videos, only printable handouts and links to online resources.

#### Control Group: American Diabetes Associations’ “Create Your Plate” Diet

This dietary intervention, our control group, was slightly different from the one we had originally used in our in-person intervention because we received feedback that the previous “carbohydrate counting” intervention was difficult for participants to follow. Instead, we randomized half of participants to a diet program based around a plate method diet, the American Diabetes Associations’ “Create Your Plate” diet, a low-fat diet that emphasizes green vegetables, lean protein sources, and somewhat limited starchy and sweet foods. All proportions are based on a 9-inch plate: half the plate is filled with nonstarchy vegetables, one-quarter with carbohydrates, and one-quarter with lean proteins [29]. We taught this group using short videos created for the study (approximately 5-10 minutes long), with printable handouts and links to online resources, such as links to online recipes and recipe books. We chose to include just the standard dietary information in this group, and not all the extra behavioral help, in order to have this condition be a minimal dietary control group. We emailed participants in this group new lessons weekly for the first 4 weeks and then every 4 weeks thereafter. This group did not get the positive affect regulation and mindful eating materials.

All participants in both groups could contact the first author by phone or email as needed with questions. A coauthor (SK) was on-call by pager for any urgent medical concerns. All regularly planned emails were sent automatically by custom software, which allowed us to ensure that the emails arrived to participants in a timely manner and removed the need for study staff to oversee this process.

### Measures

All measures were assessed at baseline before randomization and at 16 and 32 weeks after the intervention began.

#### Metabolic Measures

We assessed HbA_1c_ as well as fasted low- and high-density lipoprotein cholesterol (LDL and HDL) and triglycerides at a commercial Clinical Laboratory Improvement Amendments-certified laboratory (LabCorp; Laboratory Corporation of America Holdings, Burlington, NC, USA).

#### Body Weight

Participants recruited at the start of the study had their body weight measured at a US HealthWorks Medical Group (Valencia, CA, USA) location, near wherever they lived. Due to measurement concerns (eg, participants were asked their weight instead of actually being weighed), we then mailed the participants the EatSmart Digital Bathroom Scale. At each critical time point, participants emailed study staff a photo of their feet and digitally displayed weight while they stood on the digital scale.

#### Psychological Self-Report

Participants completed the Diabetes Distress Scale [30], a measure of upset related to having diabetes. We assessed the subjective experience of each diet by asking, “How much do you like how you feel on your diet?” and “How much do you think your diet has improved your physical health?” all rated from 1 (not at all) to 7 (very much so). We further asked, “How often do you cheat on your diet?” rated from 1 (not at all) to 7 (very often) and “How hard is it to stay on your diet?” rated from 1 (not at all) to 7 (very difficult).

We measured depressive symptoms with a 20-item scale, the Center for Epidemiologic Studies Depression Scale (CESD) [31], with higher scores reflecting greater symptoms over the past week. Following past research, we also separately examined four items that tap into positive affect, including “I felt hopeful about the future” and “I was happy.” Higher scores reflect greater positive affect.

Participants completed the Modified Differential Emotions Scale (mDES) [32], which gauges negative and positive mood. This version of the mDES asked participants to recall the past week and rate how often they had experienced particular emotions, rated from 1 (not at all) to 9 (all the time). The positive emotions subscale consists of amusement, awe, compassion, contentment, gratitude, hope, interest, happy, love, and pride. The negative emotions subscale consists of anger, contempt, disgust, embarrassment, anxiety, guilt, sadness, boredom, and loneliness.

#### Physical Self-Report

We assessed physical symptoms with an adaptation of the Health Symptom Checklist, a short, face-valid measure of physical symptoms [33], rated from 1 (not at all) to 4 (very often) for how often over the past week they had experienced a variety of physical symptoms. We used a subscale of the Short Form Health Survey [34], a well-validated and extensively used measure of health-related quality of life, to assess vitality (energy and fatigue).

#### Dietary Self-Report

We assessed dietary composition using the free online application MyFitnessPal [35], which has a vast database of foods and has been or is being used in other clinical trials [36-38]. Even so, its database is partially user-generated and results may be prone to error. Therefore, the dietary self-report results should not be considered validated. At each of the main time points, participants reported on what they had eaten over two weekdays and one weekend day, which we then averaged into one composite measure.

#### Statistical Analyses

The primary statistical analyses were performed using Stata IC software version 14.1 (StataCorp LP, College Station, TX, USA) setting two-tailed alpha to reject the null hypothesis at .05. Our experimental design randomized participants to one of two groups (intervention: n=12; control: n=13) participating in a 32-week online dietary and lifestyle intervention designed for weight and HbA_1c_ reductions with primary outcomes (HbA_1c_ and body weight) measured at three time points (baseline, week 16, week 32). All our main outcomes were continuously scaled and were appropriately analyzed with parametric statistical techniques, and all statistical assumptions were tested prior to interpreting results. The data met the distributional requirements for the techniques employed without requiring data transformations, model adjustments (eg, random slope terms, heteroscedasticity adjustments), or nonlinear modeling.

Participants’ repeated measures outcomes were submitted to separate mixed-effects linear regression analyses with fixed effect terms comparing baseline (preintervention) to each of the two subsequent observations made postintervention (weeks 16 and 32), the main effect for group, and most importantly, the simple interaction effects comparing the relative change by group at each postintervention assessment, relative to baseline. Random y-intercept terms were included to accommodate for the repeated measures experimental design. Our analysis of total caloric intake, net carbohydrates, fat, and sugar required log transformations prior to analysis to normalize model residuals; out of a total possible 63 observations, we eliminated one triglyceride, two LDL, and one calorie observations that were overly influential outliers.

For all the self-reported ratings of the subjective experience of the diet, we assessed differences between the groups using Cohen *d*. For all results involving ratios, we used a two-tailed Fisher exact test to assess significance. Means and confidence intervals are reported in their original units for all variables.

## Results

**Figure 1:**
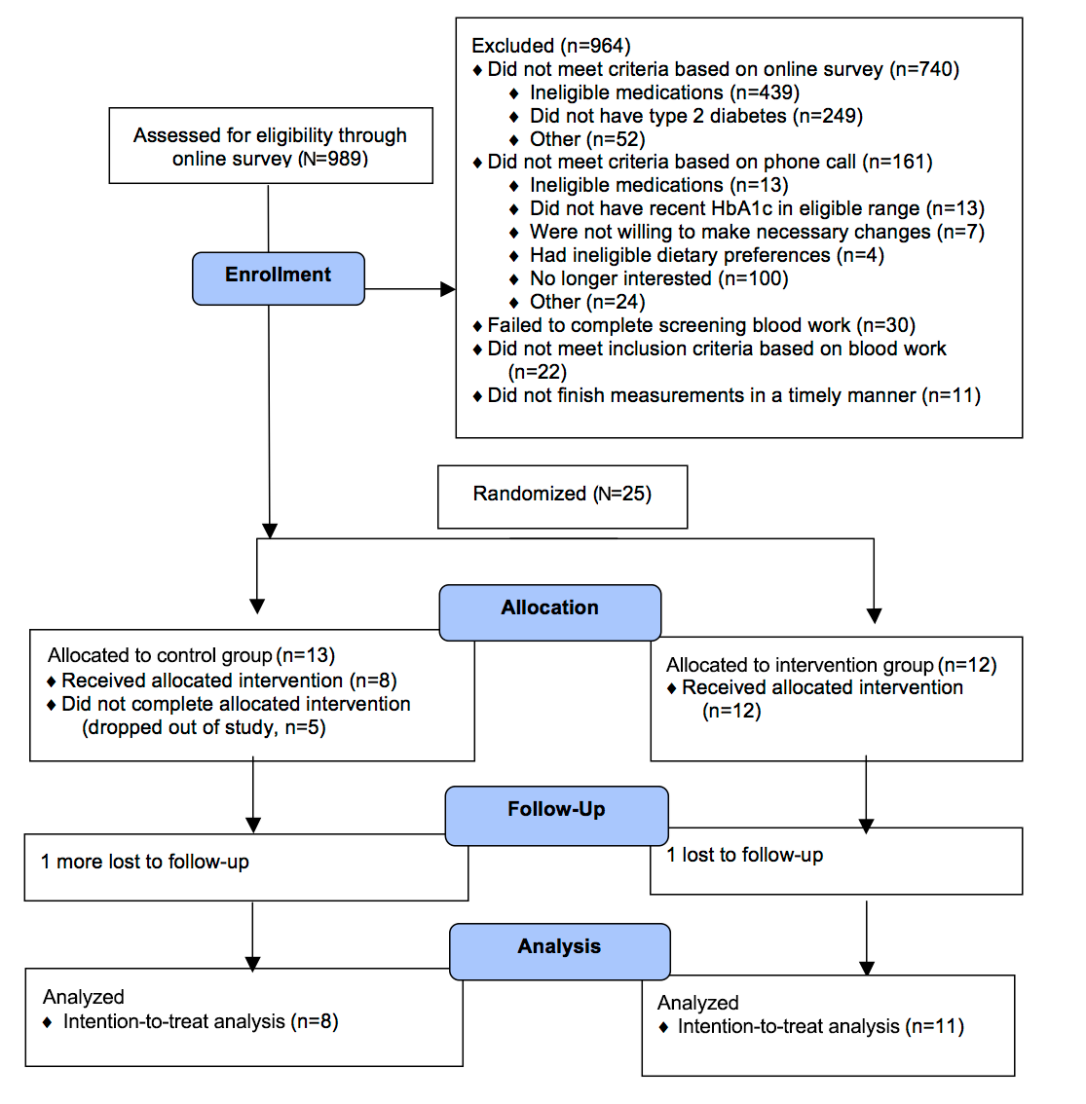
Study participant ﬂowchart for online study.

We enrolled and randomized 25 participants to the intervention (n=12) or control (n=13) group (Figure 1). A large number of individuals who took our initial online survey were ineligible because they did not have type 2 diabetes (n=249), were taking diabetes medications other than metformin (n=404), or had definite plans to begin taking insulin (n=35). Randomized participants included men and women, of several different types of ethnic and racial backgrounds (although about half were white), with an average duration of diagnosed type 2 diabetes of approximately 5 years, and a mean baseline HbA_1c_ of approximately 7% (Table 1).

**Table 1:** Baseline participant characteristics (N=25).

Baseline characteristics		Intervention group (n=12)	Control group (n=13)
**Sex, n (%)**			
	Male	6 (50)	4 (31)
	Female	6 (50)	9 (69)
Age (years), mean (SD)		53.0 (10.2)	58.2 (6.7)
**Race/Ethnicity, n (%)**			
	Asian/Pacific Islander	2 (17)	2 (15)
	Black	3 (25)	0 (0)
	White	7 (58)	8 (62)
	Latino/a	2 (17)	5 (38)
Duration of diabetes (years), mean (SD)		5.3 (4.1)	5.7 (3.7)
HbA_1c_ (%), mean (SD)		7.1 (0.4)	7.2 (0.3)
Weight (kg), mean (SD)		109.7 (24.9)	90.9 (16.4)
Triglycerides (mg/dL), mean (SD)		174.1 (79.4)	151.5 (87.1)
HDL cholesterol (mg/dL), mean (SD)		45.7 (15.0)	53.9 (12.7)
LDL cholesterol (mg/dL), mean (SD)		96.9 (30.4)	90.5 (29.0)
Diabetes-related distress, mean (SD)		1.9 (0.8)	2.4 (1.2)
CES-Depression, mean (SD)		10.5 (7.7)	9.8 (7.4)
CES-D Positive Affect, mean (SD)		10.2 (2.3)	10.2 (2.2)
DES Negative Affect, mean (SD)		2.8 (1.3)	2.7 (1.4)
DES Positive Affect, mean (SD)		6.5 (1.1)	6.2 (1.5)
Vitality (SF-36 subscale), mean (SD)		53.3 (16.4)	49.2 (20.1)
Total kilocalories, mean (SD)		1768.5 (626.6)	1749.1 (322.2)
Total grams of nonfiber carbohydrates, mean (SD)		163.6 (86.7)	152.0 (58.9)
Total grams of fat, mean (SD)		77.1 (41.4)	81.3 (27.3)
Total grams of protein, mean (SD)		83.3 (18.0)	74.5 (17.2)
Total grams of sugar, mean (SD)		50.6 (33.8)	44.9 (23.8)

Trial retention differed by group. Dropout was higher in the control group: 16-week dropout for the intervention group was zero of 12 (0%) and 5 of 13 (39%) for the control group (*P*=.04); 32-week dropout for the intervention group was 1 of 12 (8%) and 6 of 13 (46%) for the control group (*P*=.07). One participant in each group reported experiencing an event that they believed was caused by hypoglycemia (one in the control group was likely from eating very few calories and the other in the intervention group was after taking a dose of metformin).

### Metabolic Measures

#### Glycated Hemoglobin

**Figure 2:**
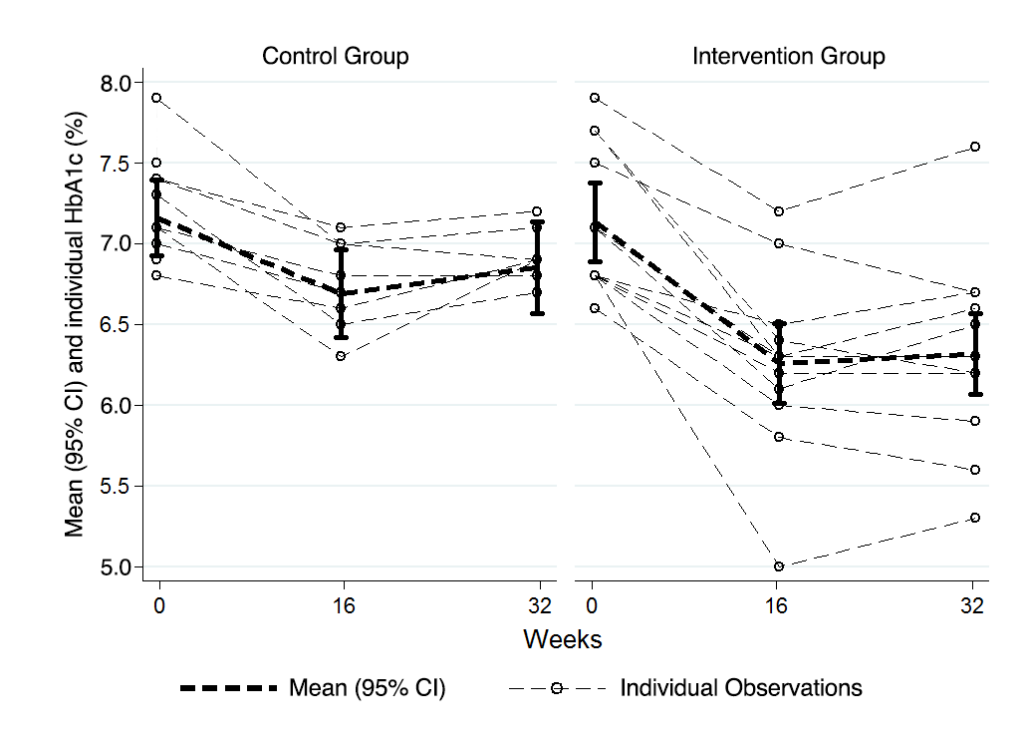
Mean and individual body weight (in kilograms) for the intervention and control groups at baseline and at 16 and 32 weeks. Bars represent 95% confidence intervals of the mean. Dashed lines reflect individual participants; darker lines represent each group mean.

There were significantly greater reductions in HbA_1c_ for the intervention group relative to the control group at both 16 (*P*=.01) and 32 (*P*=.002) weeks. Reductions in HbA_1c_ were approximately twice as large in the intervention versus the control group (intervention group: estimated marginal mean [EMM] –0.9% at 16 weeks and EMM –0.8% at 32 weeks; control group: EMM –0.5% at 16 weeks and EMM –0.4% at 32 weeks; Table 2,Figure 2). At both 16 and 32 weeks, a greater percentage of participants in the intervention group lowered their HbA_1c_ to less than 6.5%, the cutoff for type 2 diabetes, compared to the percentage of participants in the control group (intervention group: 9/12, 75% at 16 weeks and 6/11, 55% at 32 weeks; control group: 1/8, 13% at 16 weeks and 0/8, 0% at 32 weeks; Table 3). We redid these analyses using participants’ baseline body weight as covariates in the model. The results were nearly identical; therefore, we present the simpler unadjusted model.

**Table 2:** Estimated marginal mean (EMM) changes from baseline to 16 and 32 weeks.^a^

Outcomes		Intervention group, EMM (95% CI)^b^	Control group, EMM (95% CI)^c^	Difference between groups, EMM (95% CI)	*P*
**HbA_1c_ (%)**					
	16 weeks	–0.9 (–1.1, –0.6)	–0.5 (–0.8, –0.2)	–0.4 (–0.7, –0.1)	.01
	32 weeks	–0.8 (–1.1, –0.6)	–0.3 (–0.6, 0.0)	–0.5 (–0.8, –0.2)	.002
**Weight (kg)**					
	16 weeks	–8.5 (–11.9, –5.2)	–3.9 (–8.0, 0.2)	–4.6 (–8.8, –0.4)	.03
	32 weeks	–12.7 (–16.1, –9.2)	–3.0 (–7.3, 1.3)	–9.6 (–14.0, –5.3)	<.001
**Triglycerides (mg/dL)**					
	16 weeks	–35.5 (–65.7, –5.2)	–17.4 (–55.2, 20.4)	–18.1 (–56.1, 19.9)	.35
	32 weeks	–60.1 (–91.3, –28.9)	–6.2 (–46.0, 33.6)	–53.9 (–93.6, –14.2)	.01
**HDL cholesterol (mg/dL)**					
	16 weeks	1.4 (–2.7, 5.6)	–0.3 (–5.3, 4.8)	1.7 (–3.4, 6.8)	.52
	32 weeks	4.8 (0.5, 9.1)	0.6 (–4.7, 5.9)	4.1 (–1.2, 9.5)	.13
**LDL cholesterol (mg/dL)**					
	16 weeks	–0.8 (–10.9, 9.4)	1.5 (–11.7, 14.7)	–2.2 (–15.3, 10.8)	.74
	32 weeks	–0.3 (–10.8, 10.3)	–6.1 (–19.9, 7.7)	5.9 (–7.8, 19.5)	.40
**Diabetes-related distress**					
	16 weeks	–0.5 (–0.8, –0.1)	–0.3 (–0.7, 0.1)	–0.1 (–0.6, 0.3)	.49
	32 weeks	–0.4 (–0.8, 0.0)	–0.4 (–0.8, 0.0)	0.0 (–0.5, 0.5)	.98
**CES-Depression**					
	16 weeks	–3.7 (–7.8, 0.5)	0.8 (–3.7, 5.4)	–4.5 (–9.3, 0.4)	.07
	32 weeks	–0.6 (–5.0, 3.7)	–1.0 (–6.0, 4.0)	–0.4 (–4.8, 5.6)	.88
**CESD Positive Affect**					
	16 weeks	8.4 (–5.3, 22.2)	–4.4 (–19.1, 10.3)	12.9 (–2.9, 28.7)	.11
	32 weeks	0.5 (–13.6, 14.8)	7.2 (–9.0, 23.4)	–6.6 (–23.5, 10.3)	.45
**DES Negative Affect**					
	16 weeks	–0.7 (1.5, 0.1)	–0.1 (–0.9, 0.8)	–0.6 (–1.5, 0.3)	.19
	32 weeks	–0.4 (–1.2, 0.4)	–0.7 (–1.6, 0.2)	0.3 (0.5, –0.6)	.49
**DES Positive Affect**					
	16 weeks	0.5 (–0.3, 1.4)	–0.2 (–1.1, 0.7)	0.7 (–0.3, 1.7)	.15
	32 weeks	0.4 (–0.5, 1.2)	0.3 (–0.7, 1.2)	0.1 (–0.9, 1.1)	.82
**Vitality (SF-36 subscale)**					
	16 weeks	13.3 (2.5, 24.02)	2.3 (–9.4, 13.9)	11.0 (–1.4, 23.4)	.08
	32 weeks	9.2 (–1.9, 20.4)	11.0 (–1.8, 23.8)	–1.8 (–15.1, 11.6)	.80
**Total kilocalories**					
	16 weeks	–362.9 (–634.7, –91.1)	–300.8 (–594.3, –7.4)	–62.1 (–376.0, 251.7)	.65
	32 weeks	–439.3 (–719.4, –159.3)	–216.6 (–559.2, 125.9)	–222.7 (–569.9, 124.5)	.13
**Total grams of nonfiber carbohydrates**					
	16 weeks	–123.2 (–167.2, –79.2)	–27.03 (–75.4, 21.3)	–19.2 (–147.4, –44.9)	<.001
	32 weeks	–122.7 (–167.9, –77.5)	–14.8 (–71.0, 41.5)	–107.9 (–164.6, –51.3)	<.001
**Total grams of fat**					
	16 weeks	–7.8 (–36.3, 20.7)	–18.7 (–48.8, 11.4)	10.9 (–21.7, 43.4).	.42
	32 weeks	–4.0 (–33.2, 25.3)	–23.7 (–58.5, 11.1)	19.8 (–15.9, 55.5)	.13
**Total grams of protein**					
	16 weeks	–0.5 (–14.0, 13.0)	–0.2 (–15.3, 14.9)	–0.3 (–16.2, 15.6)	.97
	32 weeks	–1.6 (–15.5, 12.3)	–0.1 (–17.7, 17.6)	–1.5 (–19.2, 16.1)	.86
**Total grams of sugar**					
	16 weeks	–37.2 (–53.8, –20.5)	–4.4 (–22.9, 14.1)	–32.7 (–52.3, –13.2)	<.001
	32 weeks	–32.5 (–49.7, –15.4)	–0.3 (–21.9, 21.3)	–32.2 (–53.9, –10.6)	<.001

^a^ Data are estimated marginal means and 95% confidence intervals by linear mixed-effects model analysis.

^b^ Total analyzed in intervention group: n=12 for week 16 and n=11 for week 32.

^c^ Total analyzed in control group: n=9 for week 16 and n=8 for week 32.

**Table 3:** Percentage of people meeting HbA_1c_ and weight change thresholds.

HbA_1c_ and weight outcomes		Intervention group^a^	Control group^b^	Difference between groups	*P*
**Participants with final HbA**_1c_**<6.5%, n (%)**					
	16 weeks	9 (75%)	1 (13%)		.02
	32 weeks	6 (55%)	0 (0%)		.02
**Weight (% of initial weight), mean (SD)**					
	16 weeks	–7.8 (3.6)	–4.2 (3.7)	–3.6 (–7.1, –0.1)	.04
	32 weeks	–12.0 (7.3)	–2.5 (4.6)	–9.5 (–16.1, –2.9)	.01
**Participants achieving a 5% weight loss, n (%)**					
	16 weeks	10 (83%)	3 (38%)		.06
	32 weeks	10 (90%)	2 (29%)		.01

^a^ Total in intervention group: n=12 at 16 weeks and n=11 at 32 weeks.

^b^ Total in control group: n=8 at 16 weeks and n=8 at 32 weeks for participants with HbA_1c_ <6.5%, and n=7 at 32 weeks for participants achieving a 5% weight loss.

#### Body Weight

**Figure 3:**
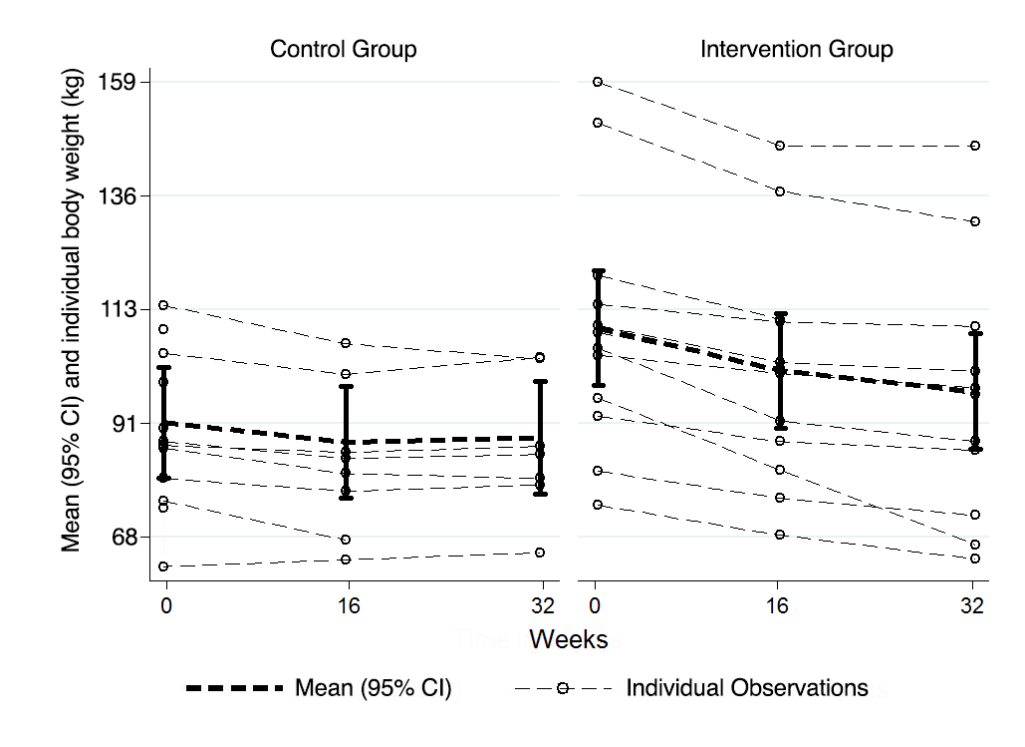
Mean and individual body weight (in kilograms) for the intervention and control groups at baseline and at 16 and 32 weeks. Bars represent 95% confidence intervals of the mean. Dashed lines reflect individual participants; darker lines represent each group mean.

We also found significantly greater reductions in body weight for participants in the intervention group relative to the control group at weeks 16 (*P*=.03) and 32 (*P*<.001). For example, at 32 weeks, participants in the intervention group lost more weight (EMM –12.7 kg, 95% CI –16.1 to –9.2 kg) than participants in the control group (EMM –3.0 kg, 95% CI –7.3 to 1.3 kg; *P*<.001) (Table 2,Figure 3). At both 16 and 32 weeks, more than double the percentage of participants in the intervention group lost at least 5% of their body weight compared participants in the control group (intervention group: 10/12, 83.3% at 16 weeks and 10/11, 90.1% at 32 weeks; control group: 3/8, 37.5% at 16 weeks and 2/8, 28.6% at 32 weeks; Table 3).

**Figure 4:**
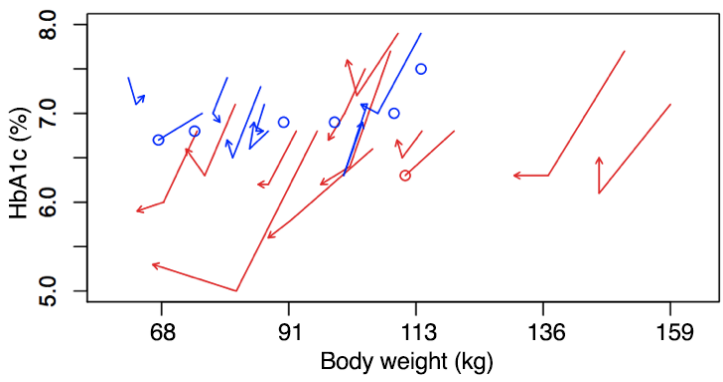
Body weight and HbA_1c_ plotted for each participant separately for each of the three time periods (0, 16, and 32 weeks). Red lines represent the intervention participants; blue lines represent the control participants. Lines that end in an O reflect dropouts (and missing data). Lines that end in an arrow show participants who completed the study.

We examined the intersection of weight and HbA_1c_ changes over time for each participant (Figure 4). Participants in the intervention group tended to show strong downward and leftward trajectories, especially from baseline to 16 weeks, reflecting a strong initial loss in weight and HbA_1c_, whereas those trajectories reflected less simultaneous change for most participants in the control group.

#### Cholesterol and Triglycerides

The intervention was also more effective at reducing triglycerides from baseline relative to the control; however, the effect was significant only at the 32-week time point (*P*=.01). Both HDL and LDL data revealed no effects between groups or differences from baseline within each group.

### Psychological Self-Report

#### Diabetes-Related Distress

We found no statistically significant effects on this measure in the intervention group relative to the control group (Table 2).

#### Depressive Symptoms, Affect, and Vitality

We found no statistically significant effects on these measures in the intervention group relative to the control group (Table 2).

#### Subjective Experience of the Diets

Compared to the control group, participants in the intervention group rated themselves as less likely to cheat on their assigned diet at 16 and 32 weeks, with a large effect size of at least a Cohen *d*=–1.0 (Table 4). Participants in the intervention group also rated their diet less difficult to stick to, better liked how they felt on the diet, and were more likely to think that their diet improved their physical health, all with medium to large effect sizes.

**Table 4:** Self-reported ratings of subjective experience of the diets.

Self-reported ratings		Intervention group, mean (SD)	Control group, mean (SD)	Cohen *d* between groups
**Overall self-rating of how much they like how they feel on the diet**				
	16 weeks	5.9 (1.1)	5.2 (1.3)	0.6
	32 weeks	6.2 (1.0)	4.9 (2.3)	0.8
**Overall self-rating of how much they think the diet improved their physical health**				
	16 weeks	6.2 (1.0)	5.3 (1.8)	0.6
	32 weeks	6.2 (0.9)	5.1 (2.5)	0.5
**Overall self-rating of likelihood of cheating on diet**				
	16 weeks	2.7 (1.4)	3.9 (0.9)	–1.0
	32 weeks	3.4 (1.1)	5.0 (0.8)	–1.7
**Overall self-rating of difficulty of staying on diet**				
	16 weeks	2.7 (1.5)	4.0 (1.3)	–0.9
	32 weeks	3.2 (1.5)	4.0 (1.7)	–0.5

### Physical Self-Report

Compared to participants in the control group, participants in the intervention group reported greater reductions in headache symptoms, bloating, and gas at week 16, as well as greater increases in constipation symptoms at week 16 (all with a large Cohen *d* effect size of at least 0.9 between groups; Multimedia Appendices 1 and 2.

### Dietary Self-Reports

At both 16 and 32 weeks, compared to the control group, the intervention group reported eating fewer grams of nonfiber carbohydrates and grams of sugar. Grams of fat and protein did not show any group effects (Table 2).

The dietary measurements suggest that participants in both groups were, on average, adherent to their assigned diet. Participants in the intervention group ate the recommended daily grams of nonfiber carbohydrates and participants in the control group ate an expected percentage of their total calories from carbohydrates. In the intervention group, daily grams of nonfiber carbohydrates lowered from a mean of 163.6 (SD 86.7) grams at baseline to a mean of 40.4 (SD 45.9) grams at 16 weeks and a mean of 43.5 (SD 33.9) grams at 32 weeks, suggesting that participants were, on average, adherent to their assigned intervention diet. Although participants in the control group were not asked to reach a particular daily target for grams of nonfiber carbohydrates, their daily intake lowered from a mean of 152.0 (SD 58.9) grams at baseline to a mean of 127.1 (SD 40.2) grams at 16 weeks and a mean of 144.8 (SD 33.7) grams at 32 weeks.

In the intervention group, the mean percentage of calories from total carbohydrates changed from a baseline mean of 39.6% (SD 10.4%) to a mean of 15.5% (SD 13.0%) at 16 weeks and a mean of 18.5% (SD 12.8%) at 32 weeks. In the control group, the mean percentage of calories from total carbohydrates changed from a baseline mean of 37.6% (SD 10.3%) to a mean of 40.9% (SD 6.3%) at 16 weeks and a mean of 43.0% (SD 9.1%) at 32 weeks. This percentage of calories from carbohydrates in the both groups suggest that participants were, on average, adherent their assigned control diet; the overall target of percentage of calories from carbohydrates was expected to be approximately less than 20% in the intervention group and about 50% in the control group.

### Medication Changes

The changes in metformin dosages were similar between groups. At 32 weeks, in the intervention group, metformin medications was decreased in one participant, increased in two participants, and unchanged in eight participants. In the control group, metformin dosage was decreased in two participants, increased in one participant, and unchanged in four participants. We had limited room to see differences in medication changes because we only enrolled participants on metformin, a drug that is safe enough and has a low enough risk of hypoglycemia that physicians do not quickly change its dosage.

## Discussion

### Principal Results

Our results show that participants randomly assigned to the very low-carbohydrate ketogenic diet and lifestyle recommendations (intervention) group had a variety of health benefits including lower HbA_1c_, body weight, and triglyceride levels, compared to those assigned to the control group (the plate method diet).

Our results are similar to those from our previous in-person trial of these very low-carbohydrate ketogenic diet and lifestyle recommendations. This online study differed from the in-person one due to its recruitment approach (national for this online study; in San Francisco, CA, for the in-person study), and allowable diabetes medications (none or just metformin for this online study; none, metformin, and/or sulfonylureas or dipeptidyl peptidase-4 inhibitor for the in-person study). These differences suggest that the online program might be applicable to overweight individuals with type 2 diabetes living across the United States, not currently taking multiple medications for their diabetes, who are motivated to make dietary changes, and have access to the Internet.

### Limitations

The ability to generalize from the results of this study is limited by its size, targeted population, and length of follow-up time. We had to screen a large number of participants in order to find eligible participants. Of those screened, 26% filled out the online survey but did not have type 2 diabetes (according to their own self-report), 47% were taking or planning to take medications that made them ineligible, 10% were not interested once they heard more about the study from the study staff online, and only a few (<1%) reported having dietary preferences counter to those on the possible assigned diets. A larger trial with a longer follow-up is needed to better understand the durability of the effects on glycemic control and weight in a broader population.

By the end of the trial, we retained 92% of participants in the intervention group, compared to 54% of the control group. This difference could have been because the intervention group’s program had more sessions and included behavioral adherence strategies, which may have made their program more engaging. In addition, glycemic control and weight loss were lower in the intervention group; some participants in the control group expressed frustration that their glycemic control or weight loss was not as much as they would like, and thus they decided to not continue with the control program. Participants in the intervention group rated themselves as less likely to cheat on their assigned diet, compared to participants in the control group. Perhaps this difference in likelihood to cheat also suggests that the intervention program was easier to adhere to (possibly due to the diet or possibly due to the extra supports included in the intervention program).

### Comparison With Prior Work

An innovative aspect of this program was remote monitoring of glycemic control, body weight, and other outcomes, suggesting that although these online program participants never met the researchers or study staff in-person, we were still able to measure and improve outcomes. Several other online interventions have successfully improved glycemic control and reduced body weight in adults, although their ability to retain participants was mixed. For example, one online program for individuals with prediabetes was based on the Diabetes Prevention Program, and it likely taught participants a lower-calorie, lower-fat diet, although this is not explicitly mentioned in the publication. After 12 months, participants’ HbA_1c_ was reduced by 0.4% and they had lost 4.8% of body weight. However, only 45% of 220 participants had follow-up HbA_1c_ values [39]. In a completely online program for overweight individuals that taught participants to follow a lower-calorie and lower-fat diet, participants in the active intervention group lost a mean 5.6% of their body weight 6 months after baseline. Of 77 participants assigned to the active intervention group, 70% were retained [40].

Not all online trials are effective. In an online self-management program for individuals with type 2 diabetes that recommended “healthy eating,” participants in the active intervention groups (with or without extra follow-up calls and visits) had not significantly reduced their HbA_1c_ levels or lost weight at 12 months after baseline. Of 331 participants assigned to either intervention group, 72% were retained [41]. In an online self-care intervention for individuals with type 2 diabetes that also recommended “healthy eating,” participants assigned to the active intervention group did not show changes in their HbA_1c_ at 6 months after baseline. Of 491 participants who began the program, 80% had 6-month outcome data [42]. Thus, the retention rate of our active intervention group (92%) was good compared to other previous online trials, as was our ability to bring about changes in glycemic control and weight in that group.

In previous trials of very low-carbohydrate diet programs in adults with type 2 diabetes that were at least 3 months or longer [7,38,43-58], researchers followed participants for a mean of 12 months. All interventions were in-person. On average, HbA_1c_ dropped 1.0% (median –0.8%). Both mean and median body weight lost was –8%. Thus, the results from our online very low-carbohydrate intervention program replicate or improve on past results, given the fact that HbA_1c_ dropped by 0.8% and body weight reduced by 12.0%.

### Conclusions

Our results lend continued support for the idea that our program’s recommendations to follow a very low-carbohydrate ketogenic diet and make lifestyle changes is promising and can bring about improved health outcomes in overweight individuals with type 2 diabetes. Future work should examine how robust these results are with larger, more diverse participants; determine whether more robust psychological or other intervention support could improve dietary adherence; track whether the positive health effects are sustained over time; and, through more thorough implementation research, whether and how such an online intervention can dovetail with existing in-person health care teams. The online delivery of this approach gives it the potential to have wider impact in the treatment of type 2 diabetes.

## Appendix

Multimedia Appendix 1 Baseline values of physical symptoms.

Multimedia Appendix 2 Change in symptoms from baseline.

## Abbreviations

CESD: Center for Epidemiologic Studies Depression Scale
HbA_1c_: glycated hemoglobin
HDL: high-density lipoprotein
LDL: low-density lipoprotein
mDES: Modified Differential Emotions Scale
