# Supplementary material for: An Online Intervention Comparing a Very Low-Carbohydrate Ketogenic Diet and Lifestyle Recommendations Versus a Plate Method Diet in Overweight Individuals With Type 2 Diabetes: A Randomized Controlled Trial
Source: J Med Internet Res. 2017 Feb 13;19(2):e36. doi: 10.2196/jmir.5806 (PMC5329646; doi:10.2196/jmir.5806)
Supplement: Multimedia Appendix 1 [file jmir_v19i2e36_app1.pdf]

Appendix Table 1. Baseline values of physical symptoms.

	Intervention group	Control group
Cold, mean (SD)	1.5 (0.8)	1.7 (1.4)
Allergy, mean (SD)	1.2 (0.4)	1.4 (0.9)
Dizziness, mean (SD)	1.7 (1.2)	1.2 (0.4)
Rapid heartbeat, mean (SD)	1.7 (1.2)	1.4 (0.9)
Short breath, mean (SD)	1.3 (0.6)	1.0 (0.0)
Ache, mean (SD)	3.0 (1.3)	2.7 (1.3)
Headaches, mean (SD)	2.1 (1.2)	1.3 (0.8)
Acne and oily skin, mean (SD)	1.4 (0.8)	1.5 (1.1)
Cramps, mean (SD)	1.8 (1.3)	1.8 (1.0)
Heartburn, mean (SD)	1.6 (0.8)	1.4 (0.8)
Constipation, mean (SD)	1.2 (0.4)	1.5 (1.0)
Diarrhea, mean (SD)	1.5 (0.7)	1.3 (0.6)
Nausea, mean (SD)	1.8 (1.2)	1.5 (0.8)
Bloat/gas, mean (SD)	2.0 (1.0)	1.7 (1.1)
