# Supplementary material for: An Online Intervention Comparing a Very Low-Carbohydrate Ketogenic Diet and Lifestyle Recommendations Versus a Plate Method Diet in Overweight Individuals With Type 2 Diabetes: A Randomized Controlled Trial
Source: J Med Internet Res. 2017 Feb 13;19(2):e36. doi: 10.2196/jmir.5806 (PMC5329646; doi:10.2196/jmir.5806)
Supplement: Multimedia Appendix 2 [file jmir_v19i2e36_app2.pdf]

Appendix table 2. Change in symptoms from baseline.

	Intervention group	Control group	Cohen's *d* between groups
Cold, mean (SD)			
16 weeks	-0.5 (0.8)	-0.7 (1.8)	0.2
32 weeks	0.1 (0.7)	0.1 (2.2)	0.0
Allergy, mean (SD)			
16 weeks	0.2 (0.8)	0.2 (1.1)	0.0
32 weeks	-0.1 (0.3)	0.4 (1.6)	-0.5
Dizziness, mean (SD)			
16 weeks	-0.2 (1.1)	0.1 (0.3)	-0.4
32 weeks	0.0 (0.5)	-0.1 (0.4)	0.3
Heartbeat, mean (SD)			
16 weeks	-0.5 (1.2)	0.0 (0.5)	-0.5
32 weeks	0.0 (0.5)	0.0 (0.6)	0.0
Short breath, mean (SD)			
16 weeks	-0.1 (0.7)	0.0 (0.0)	-0.2
32 weeks	0.3 (0.7)	0.0 (0.0)	0.6
Ache, mean (SD			
16 weeks	0.0 (1.3)	0.2 (1.2)	-0.2
32 weeks	0.1 (1.7)	-0.1 (1.1)	0.2
Headaches, mean (SD)			
16 weeks	-0.7 (0.8)	0.3 (0.5)	-1.4
32 weeks	-0.2 (0.4)	0.3 (0.5)	-1.1
Acne, mean (SD)			
16 weeks	0.0 (0.8)	-0.1 (1.8)	0.1
32 weeks	0.0 (0.0)	0.0 (1.0)	0.0
Skin, mean (SD)			
16 weeks	0.0 (0.0)	-0.3 (1.9)	0.3
32 weeks	0.0 (0.0)	-0.3 (1.3)	0.3
Cramps, mean (SD)			
16 weeks	0.4 (0.7)	0.0 (1.0)	0.4
32 weeks	0.0 (0.5)	0.1 (1.2)	-0.2
Heartburn, mean (SD)			
16 weeks	-0.3 (0.8)	0.1 (0.8)	-0.5
32 weeks	-0.1 (0.7)	-0.1 (0.4)	0.1
Constipation, mean (SD)			
16 weeks	0.7 (1.0)	0.0 (0.5)	0.9
32 weeks	0.3 (0.5)	0.0 (0.6)	0.6
Diarrhea, mean (SD)			
16 weeks	0.1 (1.2)	-0.1 (0.3)	0.2
32 weeks	-0.3 (0.5)	0.0 (0.0)	-0.9
Nausea, mean (SD)			
16 weeks	0.1 (1.2)	-0.1 (1.1)	0.2
32 weeks	0.0 (0.8)	0.1 (0.4)	-0.2
Bloat/gas, mean (SD)			
16 weeks	-0.4 (1.3)	1.2 (1.2)	-1.3
32 weeks	-0.3 (1.4)	0.9 (1.1)	-0.9
